# Levelt’s laws do not predict perception when luminance- and contrast-modulated stimuli compete during binocular rivalry

**DOI:** 10.1038/s41598-018-32703-9

**Published:** 2018-09-26

**Authors:** Jan Skerswetat, Monika A. Formankiewicz, Sarah J. Waugh

**Affiliations:** Anglia Vision Research, Department of Vision and Hearing Sciences, Anglia Ruskin University, East Road, CB1 1PT, Cambridge, UK

## Abstract

Incompatible patterns viewed by each of the two eyes can provoke binocular rivalry, a competition of perception. Levelt’s first law predicts that a highly visible stimulus will predominate over a less visible stimulus during binocular rivalry. In a behavioural study, we made a counterintuitive observation: high visibility patterns do not always predominate over low visibility patterns. Our results show that none of Levelt’s binocular rivalry laws hold when luminance-modulated (LM) patterns compete with contrast-modulated (CM) patterns. We discuss visual saliency, asymmetric feedback, and a combination of both as potential mechanisms to explain the CM versus LM findings. Competing orthogonal LM stimuli do follow Levelt’s laws, whereas only the first two laws hold for competing CM stimuli. The current results provide strong psychophysical evidence for the existence of separate processing stages for LM and CM stimuli.

## Introduction

**Figure 1:**
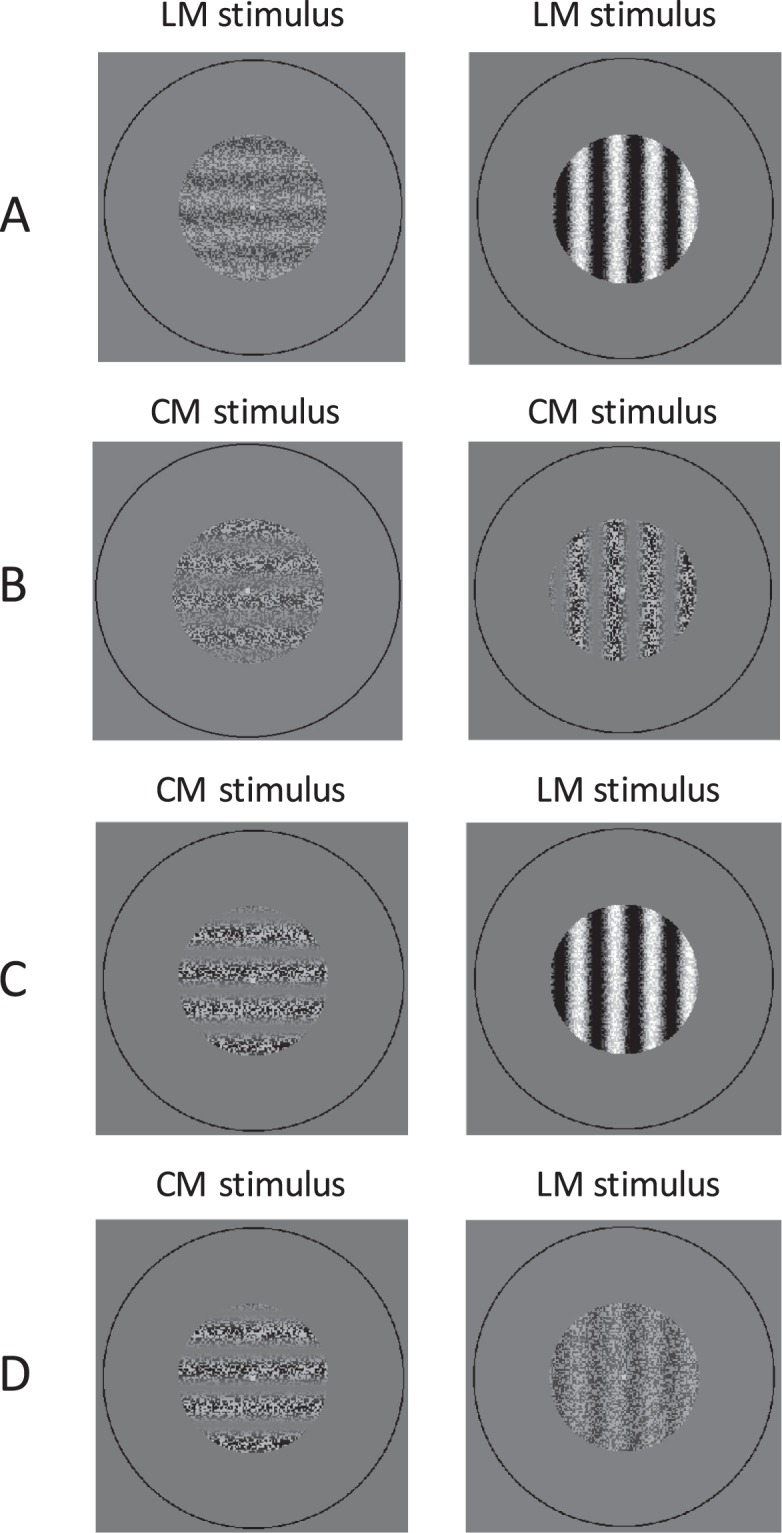
Stimuli for demonstration of binocular rivalry. (**A**) Orthogonally orientated low- and high-visibility LM gratings. (**B**) Low-visibility CM grating and a higher visibility CM grating. (**C**) Low-visibility CM grating and a high-visibility LM grating. (**D**) Similarly visible CM and LM gratings.

**Figure 2:**
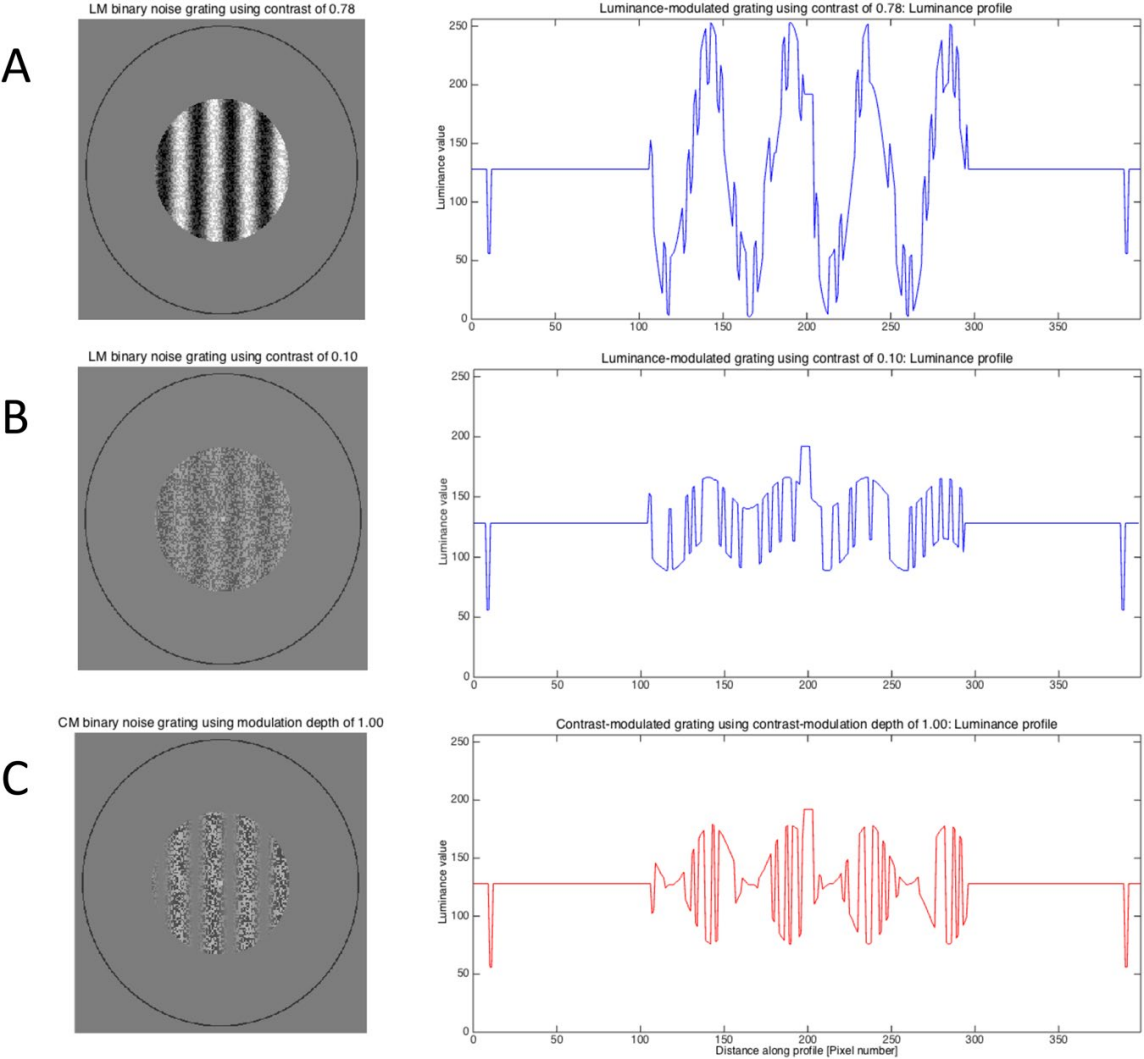
LM and CM gratings and their respective luminance profiles. High-visibility LM grating in (**A**) using contrast of 0.78. (**B**) depicts an LM grating using contrast 0.10. The variation in mean luminance follows a sine wave function. (**C**) shows a CM grating using 1.0 contrast-modulation, with no variations in overall mean luminance.

Binocular rivalry, generated by presenting incompatible stimuli separately to each eye, is a powerful tool to investigate visual perception as it can evoke alternating perception over time without any changes to the physical environment^1–3^. The reader may experience binocular rivalry by looking at the gratings in Fig. 1A held at a distance of approximately 40 cm. Position a pen tip approximately half way between the eyes and gratings, so that each eye views the pen tip centrally for one of the two gratings. Now fixate the pen tip with both eyes open. In the central patch of the now perceived three, overlapping gratings will compete perceptually. Classical laws of binocular rivalry predict that the vertical grating of high visibility will predominate perception. This relationship between stimulus visibility and perception during binocular rivalry is described by Levelt’s first law^2,4^. Levelt’s four laws are based upon findings when the competing patterns were defined by variations in luminance. Examples of similar patterns of luminance-modulated noise (LM), or first-order gratings, are shown in Fig. 2A,B.

After the publication of his thesis, Levelt’s laws were the subject of many studies^5–7^, the results of which led to some modifications to the original laws. A recent review^2^ updated Levelt’s laws to take into account the current state of knowledge. Levelt’s modified four laws are:

- “I. Increasing stimulus strength for one eye will increase the perceptual predominance of that eye’s stimulus.
- II. Increasing the difference in stimulus strength between the two eyes will primarily act to increase the average perceptual dominance duration of the stronger stimulus.
- III. Increasing the difference in stimulus strength between the two eyes will reduce the perceptual alternation rate.
- IV. Increasing stimulus strength in both eyes while keeping it equal between eyes will generally increase the perceptual alternation rate, but this effect may reverse at near-threshold stimulus strengths.” (Brascamp *et al*. 2015, page 27)

The first, second, and third laws as stated above describe the effects of varying interocular differences in stimulus strength, for example by changing the contrast of one stimulus, whereas the fourth law describes the impact of binocular stimulus strength variations.

Visual stimuli can also be created using variations of contrast, such as contrast-modulated (CM) or second-order gratings, across which the mean luminance does not change^8^ (Fig. 2C). Rivalry competition that occurs between equally visible orthogonal CM gratings, leads to fewer moments of exclusive visibility of the rival gratings and greater proportions of superimposed percepts, compared to when LM gratings are used^9,10^. As for LM gratings, when a more visible CM vertical grating rivals with a less visible horizontal CM grating, the vertical grating will predominate perception (Fig. 1B).

When the reader initiates rivalry with competing LM and CM gratings of different visual strengths, what happens? By viewing Fig. 1C, the reader is likely to perceive that the proportion of time spent seeing exclusively either grating, will be approximately the same. This would seem remarkable as the visibility of the right LM grating is much stronger compared to the less visible left CM grating. Levelt’s first law predicts that the grating on the right should predominate perception across time. The visible strength of the LM grating on the right is lowered in Fig. 1D to be approximately equal in strength to the orthogonal CM grating on the left. When completely overlapped with the aid of the technique described above, the left CM grating now strongly predominates perception. This finding is not expected according to Levelt’s first law of binocular rivalry.

The main aim of the current study was to systematically test whether rivalrous LM and CM gratings obey Levelt’s laws. In the first experiment, a CM grating is presented to one eye and an orthogonally orientated LM grating to the other (i.e. *CM vs*. *LM experiment*). The contrast (and thus visibility level) of the CM grating was fixed (at 7x detection threshold), but the visibility of the LM gratings varied (from 2x to 43x above detection threshold). Visibility levels were determined by an approximation of multiples over detection threshold from a previous study^10^. For the second experiment, visibility levels for competing orthogonally oriented CM stimuli were varied (from 2 to 7x detection threshold) to test Levelt’s laws. Characteristics of rivalry were compared to those generated using similarly visible rivalrous LM stimuli (i.e. the *CM vs*. *CM and LM vs*. *LM experiment*). The insights gained by investigating binocular rivalry when LM and CM stimuli compete with each other in particular, contribute to the ongoing debate of whether visual competition arises early, later or perhaps in multiple visual areas of the brain^11–13^.

## General Methods

The stimuli were two circular grating patches drawn on right and left sides of the screen with a mean luminance background of 49.8 cd/m^2^. A four-mirror stereoscope composed of optical components (OptoSigma Corporation, California, USA) was used for combining the stimuli, presented one to each eye. The effective viewing distance through the mirror stereoscope was 100 cm. At this distance, a screen pixel subtended 1.3 arcmin. The stimulus size in diameter was 2 deg containing a 2c/deg sinusoidal grating. The surrounding circular annuli provided a fusion lock (diameter of 4 deg and a width of 2.6 arcmin). Interocularly correlated noise (i.e. noise pixels corresponding in space, time, and luminance) was used. For both LM and CM gratings, binary noise was presented dynamically to prevent pixel clumping, which can create consistent luminance (i.e. first-order) cues in the CM (i.e. second-order) gratings if static noise is used^14^. Cycling of ten stimulus pages every 2 frames (14.3 ms) generated the dynamic noise.

Each noise check size was 2 × 2 pixels in size. The stimuli were presented on a Mitsubishi Diamond Pro 2070SB CRT monitor with a spatial resolution of 1027 × 769 pixels and a temporal frame rate of 140 Hz. A customised MatLab program in combination with the Cambridge Research Systems Visual Stimulus Generator was used to create the stimuli, run the experiment, and store the data.

Careful monitor calibration prior to stimulus presentation are essential for investigations using second-order stimuli. Gamma correction linearized luminance characteristics against voltage, using a Cambridge Research Systems ColorCal and software. Adjacent pixel non-linearity may also confound a second-order signal producing local first-order artefacts^15,16^. To minimise these effects, two pixel noise checks were used^17^.

Participants indicated their perception via a response box. They indicated whether they perceived an exclusively visible horizontal or vertical grating, piecemeal, or superimposed percepts. All experiments were performed in a dark room. Each participant sat on a comfortable chair and placed their head on chin and forehead rests so that they were comfortable when viewing stimuli through the stereoscope. Before an experiment began, the stimuli were aligned by adjusting the position of dichoptically viewed nonious markers to ensure comfortable binocular viewing through the stereoscope.

Ethics approval to conduct the experiments on human participants was obtained from the Faculty of Science and Technology Research Ethics Panel (FST/FREP/12/327) at Anglia Ruskin University, in line with the ethical principles of the Helsinki declaration of 1975. All participants were provided with written and verbal information about the project in advance, gave written informed consent before taking part, and were reimbursed for time spent.

### Experimental participants

#### CM vs. LM experiment

Levelt’s first three laws of binocular rivalry were investigated for LM and CM gratings presented to different eyes in 10 binocularly normal adults: five male and five female participants with an average age of 22.9 years (± 4.3 standard deviation). All except one participant (author J.S.) were naïve to the purpose of the study. All participants had normal or corrected-to-normal vision of at least 6/6 and normal binocular vision indicated by a measure of 60 arcsec or better, using the Dutch Organization for Applied Scientific Research (TNO) test for stereoscopic vision (Lameris Ootech, Ede, Netherlands). To test the fourth law for CM vs LM stimuli, five adults (3 male) participated.

#### LM vs. LM and CM vs. CM experiments

Four female and six male participants (six participants from the CM vs. LM experiment) of an average age of 26 ± 6.2 years participated in the LM vs. LM, and CM vs. CM experiments. A control experiment for testing the third law for LM vs LM and CM vs CM conditions, included four participants (3 female) of an average age of 27 ± 3.3 years. All inclusion criteria were the same as noted above for participants in the CM vs. LM experiment.

### Stimuli

The stimulus types can be mathematically described as follows.

Sinusoidal luminance-modulated (LM) grating:

$${l}_{0}(x,y)={l}_{0}[1+nN(x,y)+lsin(2\pi x{f}_{x})]$$

Two-dimensional binary white noise added to a sinusoidal luminance grating. *N* is the binary noise at position (*x*, *y*) (either black (−1) or white (1)) and *n* has a contrast of 0.2.

Sinusoidal contrast-modulated (CM) grating:

$${l}_{0}(x,y)={l}_{0}[1+nN(x,y)+nN(x,y)msin(2\pi x{f}_{x})]$$

Contrast modulation is *m*. The mathematical term $nN(x,\,y)msin(2\pi x{f}_{x})$ expresses the contrast-modulated grating that results from the multiplying random noise sample by a sinusoid^15^.

#### CM vs. LM experiment

In order to test the first three laws, the visibility of the CM grating was fixed at peak physical modulation (of 1.0), while the visibility of the LM grating was varied. LM gratings were created by adding dynamic two-dimensional binary noise with an amplitude of 0.2, to the sine wave (maximum modulation of 0.78). The same noise amplitude was multiplied by the sine wave to create the CM gratings with a modulation of 1.0 and was perceived at a visibility level (multiples over detection threshold) of 7 ± 1 (SEM) times. The LM contrasts were 0.04, 0.10, 0.20, 0.40 and 0.78 with perceived visibilities of approximately 2 ± 1, 5 ± 1, 11 ± 2, 22 ± 3 and 43 ± 6, respectively. These LM and CM grating visibility levels were determined in a previous study^9^. The participants of that study^9^ were asked to indicate whether a grating (having the same characteristics as those used in the current study) was detected in the first, or second, 500 ms temporal interval, while the fellow eye viewed a mean luminance screen. A method of constant stimuli combined with eleven modulation levels separated by 1.5 dB steps, allowed psychometric functions to be generated. Detection thresholds were calculated from Weibull functions fit to detectability data.

To test Levelt’s fourth law, visibilities of gratings presented to each eye were set to be the same at ~3.5 × , ~5x or ~7x above detection threshold. Luminance-modulations of 0.07, 0.10, 0.14 and contrast-modulations of 0.50, 0.70, 1.00 were used for LM and CM stimuli, respectively, to achieve these visibility levels.

#### LM vs. LM and CM vs. CM experiments

To test the first three laws, the visibility for the fixed stimulus was 3.5x. The visibility of the grating to the other eye was either 2x, 3.5x, and 7x detection thresholds. These levels were generated using the following modulations: for the LM gratings, the fixed modulation was 0.07 whilst the variable ones were 0.04, 0.07 and 0.14; for the CM gratings, fixed modulation was 0.50 whereas the variable modulations were 0.28, 0.50 and 1.00. To increase the visibility range more for LM stimuli, 0.07 and 0.78 LM contrast (3.5x and 43x above detection thresholds, respectively) were included. For the fourth law, we presented equally visible LM and CM gratings of 3.5x detection threshold. Data for LM gratings 22x above detection threshold as well as for CM gratings (7x detection threshold) were taken from a previous study^10^.

### Experimental order and analysis

In all experiments, one trial constituted data being recorded for 120 seconds. Instructions and practice trials were given before data collection began. Breaks in-between trials were permitted. A customized Matlab program was used to analyse raw data. Repeated measures ANOVAs with Greenhouse-Geisser corrections and planned comparisons were carried out using Statistica (Stat Soft, Int., USA).

Proportions of exclusive visibility perceived for each stimulus, their respective mean durations and full flip rates (abrupt changes from one exclusively visible percept to the other without mixed percepts in between) were calculated and then averaged across trials and participants to test Levelt’s first three laws for these two types of stimulus. As Levelt’s fourth law involves alternation rates, only full flip rates were calculated and averaged across trials and participants.

#### CM vs. LM experiment

To test the first three laws, an experiment for one participant comprised two sessions, each carried out on separate days. The experimental order of a session was counterbalanced in respect to grating orientation, visibility, and stimulus type-to-eye relation. One session lasted between 45 and 60 min, depending on breaks taken by each participant. To test the fourth law, an experiment was carried out in one day and included 8 trials per LM/CM combination as described in *2*.*2 Stimuli*, resulting in 24 trials in total. This experiment took around 60 minutes.

#### LM vs. LM and CM vs. CM experiments

In the second part of the current study, Levelt’s four laws were also tested when CM-CM and LM-LM gratings competed. To clarify, two orthogonal gratings of the same type; either LM vs. LM or CM vs. CM were presented to each eye. This experiment was carried out in two sessions on separate days. One session included three conditions (i.e. variable either 2x, 3.5x or 7x vs. fixed 7x), each was repeated 8 times, resulting in 24 trials and took approximately 60 minutes. The experimental order of a session was counterbalanced in respect to grating orientation, visibility and stimulus type. For the analysis of the fourth law, we compared the results of full flip rates when both types of gratings were presented at the same visibility of 3.5x, with findings from a previous study^10^. In this previous study, binocular rivalry was initiated with LM-LM gratings at a 22x visibility level and CM-CM gratings at visibility levels of 7x, but had otherwise the same properties as the stimuli used in the current study.

## Results

### Levelt’s first law

**Figure 3:**
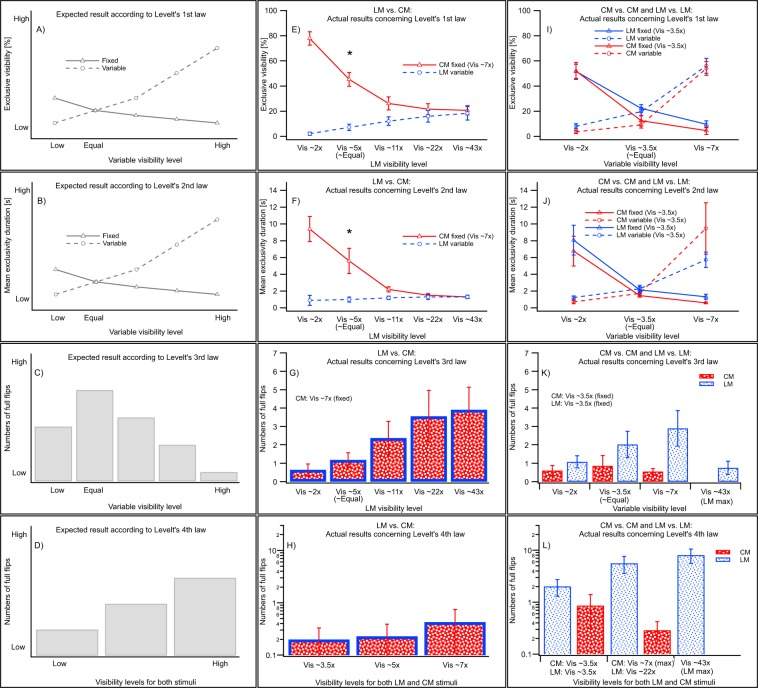
Comparison of Levelt’s four laws and the results of the current study. Visibility serves as metric to indicate stimuli strengths (x-axes). The graphs in the left column (**A**–**D**) indicate the expected results following Levelt’s laws; the graphs in the middle column (**E**–**H**) depict the actual CM vs. LM results; the graphs in the right column (**I**–**L**) depict the actual CM vs. CM and LM vs. LM results. The asterisk represent a significant difference (p < 0.05).Vertical bars represent ± 1 SEM.

Levelt’s first law predicts that a grating of higher visibility will predominate perception (Fig. 3A).

#### CM vs. LM experiment

When a high-visibility LM grating is presented to one eye and a low-visibility CM orthogonal grating is presented to the other, this prediction of perception fails. The proportion of time for exclusivity of the LM grating was never greater than for the CM grating (Fig. 3E). This counterintuitive finding is the key result of the current study. When a highly visible LM grating (43x) competes with a CM grating of much lower visibility (7x), the proportion of time for which exclusive visibility of CM or LM gratings is not significantly different [F(1,9) = 0.3, *p* > 0.05, planned comparisons]. When the two gratings are of similar visibility level (~5 to 7x), the CM grating predominates perception (i.e., it is perceived exclusively for a greater proportion of time than is the LM grating) [F(1,9) = 50.6, *p* < 0.001]. The asterisk on Fig. 3E indicates where statistical differences begin (p < 0.05).

#### CM vs. CM and LM vs. LM experiments

The results for competing LM-LM and CM-LM gratings follow the predicted pattern according to Levelt’s first law (Fig. 3I). The reader may experience these results by free-fusing the gratings (for instructions, see introduction) in Fig. 1B.

### Levelt’s second law

The second law predicts that mean exclusivity duration of the stronger stimulus will increase as the difference in visibility between the rival stimuli increases, whereas mean exclusivity duration of the weaker stimulus will decrease slightly (Fig. 3B).

#### CM vs. LM experiment

When a high-visibility LM grating is presented to one eye and a much lower visibility CM orthogonal grating is presented to the other, the prediction of the second law fails. The mean exclusivity durations are not significantly different [F(1,9) = 0.1, *p* > 0.05], even when the difference in visibility levels between the LM (at 43x) and CM (at 7x) was greatest (Fig. 3F). Moreover, the difference in mean exclusivity durations increases with decreasing visibility of the LM grating. The mean exclusivity duration of the LM stimulus did decrease, as predicted, when its visibility level is reduced (1.3 ± 0.2 s to 0.9 ± 0.6 s, LM (43x) to LM (2x), respectively), although this trend did not reach statistical significance [F(1,9) = 0.7, *p* > 0.05]. Again, the asterisk on Fig. 3F indicates where statistical differences begin at p < 0.05.

#### CM vs. CM and LM vs. LM experiments

The results for LM and CM stimuli follow the pattern as predicted by Levelt’s second law (Fig. 3J).

### Levelt’s third law

The third law predicts that the flip rate should be highest when visibility levels of two rivalling stimuli are the same. Flip rate should reduce as the difference between the visibility levels increases (Fig. 3C).

#### CM vs. LM experiment

Experimental results do not follow this prediction when CM and LM gratings compete, but rather follow a different pattern (Fig. 3G). Full flips occur most often when the difference in visibility levels between the LM and CM stimulus is greatest (3.9 ± 1.2 Full flips/trial). They occur less often as the difference in visibility levels decreases. For approximately equal visibility gratings (for LM 5x; CM 7x) there are 1.2 ± 0.4 Full flips/trial.

#### CM vs. CM and LM vs. LM experiments

Competing CM-CM gratings show a mild but not significant trend towards the expected pattern of results, i.e. full flip rate for CM (3.5x) condition was not significantly greater than for CM (2x) [F(1,9) = 0.6, *p* > 0.05] and CM (7x) [F(1,9) = 0.4, *p* > 0.05]. Note that the mean flip rate is never greater than 1 during the 120 s trial. For LM stimuli, the full flip rate is highest when measured using 7x visibility level in one eye and 3.5x visibility in the other (2.9 ± 1 Full flips/trial), which is slightly at odds with the third law. This may reflect that the visibility levels are estimated (Fig. 3K). For LM stimuli of 3.5x and 43x visibility level, does show a decrease in full flip rate (0.8 ± 0.4 Full flip/trial) is shown that is comparable to the 2x and 3.5x(1.1 ± 0.3 Full flip/trials) condition, in line with Levelt’s third law.

### Levelt’s fourth law

The fourth law predicts that the flip rate should increase when increasing the visibility levels of two rivalling, but equally visible stimuli (Fig. 3D).

#### CM vs. LM experiment

As for the other three laws, this prediction fails when increasing the visibility equally for LM and CM stimuli (Fig. 3H). In fact, full flips were almost absent (see y-axis) and although the number increased with change in visibility (from 3.5 to 7x) this was not significant [F(1,4) = 1.3, *p* > 0.05]. We will address this mild, but not significant increase of flip rate in the discussion.

#### CM vs. CM and LM vs. LM experiments

The full flip rate for LM gratings increases significantly [F(1,4) = 23.4, *p* < 0.05] with increases in visibility level, but is not significantly different [F(1,4) = 1.3, *p* > 0.05] for CM gratings (Fig. 3L). Although the visibility levels for CM gratings are relatively low, we suggest in the discussion, why it is unlikely that the fourth law would hold even if hypothetically higher visibility levels could be tested for CM vs. CM gratings.

## Discussion

### Why do highly visible LM stimuli not predominate perception over less visible CM stimuli?

**Figure 4:**
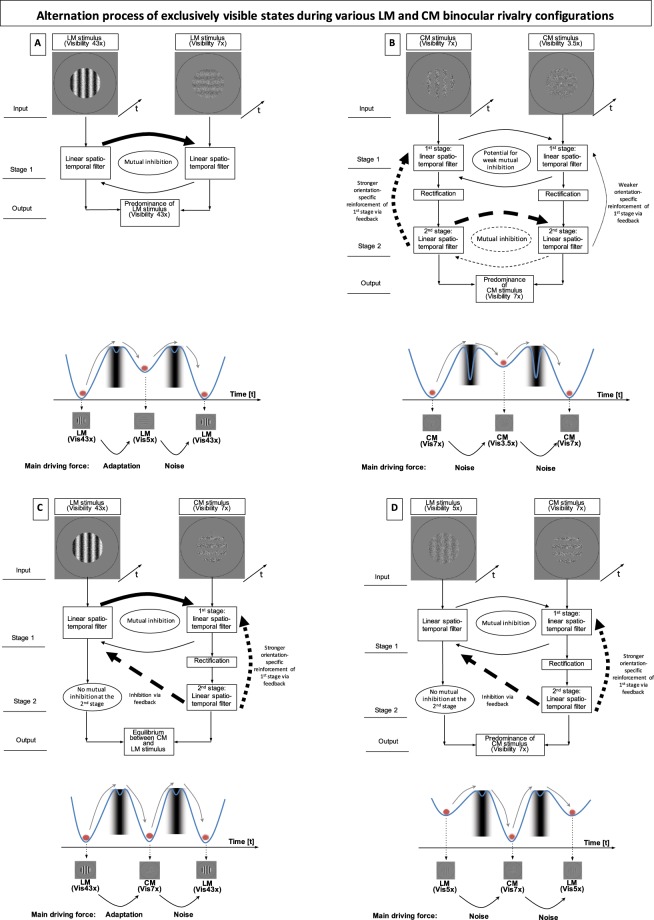
Conceptual frameworks for LM and CM binocular rivalry. Single (**A**) and two-stage (**B**–**D**) frameworks show key initial processing stages involved and indicate roles of saliency and asymmetric feedback for mediating alternations between exclusive percepts. Below each framework are energy landscape model schematics^9^ to show how forces move perception from one exclusive state, through transition states (i.e. piecemeal and superimposition) depicted as graduations of grey levels, to the other exclusive state. (**A**) Orthogonally orientated high- and low-visibility LM gratings. (**B**) High-visibility CM grating and a lower visibility CM grating. (**C**) High-visibility LM grating and a lower visibility CM grating. (**D**) Similarly visible LM and CM gratings.

Figure 4 shows schematics of different frameworks for understanding rivalry processing mechanisms. Binocular rivalry is thought to result from mutual inhibition between monocular neurones^12^ (Fig. 4A).

We demonstrated in a previous study that orthogonal CM gratings in competition with each other generate significantly greater proportions of perceived superimposition, compared to that generated by equally visible LM gratings^10^. This finding suggests that CM stimuli are processed by neurones that initially combine information from the two eyes^18,19^. The results of the current study showed that competing CM gratings of different visibilities follow Levelt’s first law (Fig. 3I). CM stimulus processing has been described using a ‘filter-rectify-filter’ model^8,15,20^. The first filter stage computes the luminance information of noise pages of the rivalling stimuli, which are spatially and temporally correlated, but may contain slightly different luminance values as different orientations must be created. This slight difference in luminance input has the potential to cause weak mutual inhibition between the noise pages of the competing CM stimuli (Fig. 4B, R-L and L-R thin curved arrows), however, the difference seems too small to generate the overall predominance of the stronger CM stimulus.

Orthogonal orientations extracted at the second filter stage are more likely to be the source of mutual inhibition, explaining why the more highly visible CM stimulus predominates perception (Fig. 4B, L-R thick dashed curved arrow) over the less visible CM stimulus (Fig. 4B, R-L thin dashed curved arrow). Studies concerning single cell recordings in primates showed that neurones, which give orientation-selective responses to second-order properties, do exist^21,22^.

In the current study, it was found (Fig. 3E) that even a highly visible LM stimulus did not predominate over a low visibility CM stimulus during binocular rivalry and that the CM stimulus dominates when both stimuli are similarly visible. Why do LM gratings never predominate perception when competing with CM gratings? These two circumstances are shown in schematics Fig. 4C,D. Three explanations are put forward to explain CM predominance during binocular rivalry: increased saliency attributed to CM stimuli, an asymmetric inhibitory feedback mechanism between stages, or potentially both saliency and asymmetric feedback contribute.

Saliency^23^ may be generated via positive feedback between each eye’s filter-rectify-filter stages (depicted as upward going dotted curves in Fig. 4B–D). In Fig. 4B, the second filter stage of the more visible CM stimulus provides stronger reinforcement of an orientation-specific response at the first stage (thick dotted line), compared to the weaker CM stimulus (thin upward dotted curve). Gradual transitions of perception between rivalling stimuli generate so-called travelling waves^23^, which have been suggested to be a mechanism for saliency^24,25^.

Another possible explanation for the CM vs. LM results is asymmetric inhibitory feedback from the higher stage of one eye’s processing pathway, to the lower stage of the other eye’s (indicated by diagonal thick arrows in Fig. 4C,D). Weak local inhibition could arise from monocular neurones at the first filter stage of the CM grating as mentioned above, however strong inhibition would arise from monocular neurones that extract the LM grating, which would lead to the prediction of strong predominance of the LM over the CM stimulus, due to its greater visibility^2,4^, which is not the case (Fig. 4E). A strong inhibitory feedback signal from binocular neurones of the second filter stage of the CM grating would be required to cause additional inhibition of the LM grating (Fig. 3E). Asymmetric, constant feedback from neurones processing CM stimuli at the second stage could provide further suppressive force (Fig. 4C,D). This would overcome LM’s ‘upper hand’ in terms of mutual inhibition at the first filter stage (Fig. 4C, thick dashed curved arrow) resulting in approximately equal proportions of exclusive visibility between the rivalling gratings. Reducing LM’s contrast further, reduces the mutual inhibition at the first stage (Fig. 4D, thin curved arrows), but since the feedback is unaffected by those changes, the CM stimulus predominates perception (Fig. 4D, thick dashed semicircle arrow).

Why would the visual system develop an asymmetric feedback mechanism? Second-order stimuli, and specifically second-stage processing, have been suggested to occur in primarily binocularity-processing neural areas^18,19,26^. The second filter stage may suppress information that disrupts binocular combination (symbolised by the dashed arrow in Fig. 4C,D). Essentially, we argue that the normal visual system favours binocular, over monocular processing mechanisms in the case of competition between CM and LM stimuli.

The existence of asymmetric interactions between first- and second-order suprathreshold stimuli has also been proposed to explain some motion aftereffects^27^, although in that study binocularly viewed, 0.5 c/deg, 1 Hz LM gratings constructed from one-dimensional noise, generated stronger motion after-effects for CM gratings, than vice versa. This asymmetry is of opposite polarity and may be due to feed-forward pooling, different to what is considered here for different stimuli viewed under binocular rivalry conditions. Our asymmetry findings are more in line with those of Schofield and Kingdom (2014)^28^ and Hairol, Formankiewicz and Waugh (2013)^29^. Schofield and Kingdom^28^ used an orientation grouping paradigm with 0.4 deg, circular patches of luminance, colour or texture (binary noise on a mean background) to assess their comparative strengths. Texture cues for orientation grouping, a task more likely based on visual saliency, dominated over luminance and colour cues. Hairol *et al*.^29^ found stronger contour interaction effects when CM bars surrounded an LM C during visual acuity assessment, than vice versa, suggesting stronger feedback effects may occur from a second CM stage. Finally, for binocularly viewed gratings (2 c/deg constructed from two-dimensional binary noise), spatial tilt and contrast after-effects for CM gratings, measured after viewing LM gratings and vice versa, were found to be similar rather than asymmetric^30^. All of the above studies used visibility-equated stimuli across stimulus types.

As stated in the introduction, controversy remains as to whether an early low-level, later high-level, or multiple processing sites of the visual pathway are responsible for binocular rivalry. Conflicting findings and ideas about how binocular rivalry is mediated and where it arises have been described elsewhere^31–33^. Our results suggest that even for the perceptual state of exclusive visibility generated by low-level grating stimuli, different neural sites within the visual cortex can be involved.

#### What causes perceptual alternations for the current stimulus conditions?

Energy landscape models incorporating a third well, which we established in a previous study^9^, help to answer this question. Each schematic in Fig. 4 involves two three-well models combined to represent three exclusivity percepts (and two transition zones) over time. Each left, middle and right well represents visual exclusivity, whereas the smaller transition wells (with grey shading) represent transitions through piecemeal and superimposed perception^9^. The red ball indicates the perceptual state shown by the stimulus pictures.

Neural adaptation^34–36^ (i.e. self-adaptation of active neurones) and intrinsic noise^37–39^ (i.e. vesicular neurotransmitter release variations, spiking variations, and fluctuations in global neurotransmitter levels^38^) have been suggested to be responsible for perceptual alternation during binocular rivalry. We suggested in a previous paper^9^ that alternations of equally visible CM gratings are more likely to be caused by intrinsic noise. Rivalry of LM stimuli for high contrast gratings is more likely caused by adaptation, whereas rivalry of low contrast gratings is more likely caused by noise^37^. These concepts are again applied for the four different stimulus conditions in Fig. 4.

The underlying neurones of the ‘stronger’ stimulus have the upper hand over the ‘weaker’ neurones because they generate stronger inhibition. Thus, a ‘stronger’ stimulus within the LM-LM (Fig. 4A) and CM-CM (Fig. 4B) processing systems will generate predominately exclusive visibility (first law) and longer mean exclusivity durations (second law). In case of the LM-LM stimulus condition (Fig. 4A), adaptation and noise contribute to rivalry, whereas for CM-CM stimulus condition (Fig. 4B) noise is likely to be the main driving cause of perceptual alternation. When a high-visibility LM grating competes with a relatively lower visibility CM grating (Fig. 4C), perceptual alternations will be triggered by both adaptation and noise. When similar low-visibility LM and CM gratings compete, the main cause for changes in perception (Fig. 4D) is noise.

#### Results in the light of Levelt’s laws

Levelt’s laws were formulated and modified for static, noiseless, luminance-based stimuli. Previous studies investigating spatial vision characteristics have proposed the existence of additional processing stages for CM compared to LM stimuli^15,18,19^. Our previous results reveal the presence of very few exclusive percepts and predominantly superimposition during binocular rivalry between competing orthogonal CM gratings, which support this idea as well^9,10^. Interocular grouping, a special case of binocular rivalry, is also thought to be mediated by a mechanism involving both binocular and monocular levels^40,41^. CM stimuli again generate mainly superimposed, rather than interocularly grouped, percepts^42^. Thus, Levelt’s laws may be restricted to interocular competition evoked within the L and LM (so-called 1^st^ order) stimulus processing stage. The current study shows that when CM stimuli interocularly compete with LM stimuli, the dynamics of binocular rivalry are not predicted correctly by Levelt’s four laws (Fig. 3E–H). When CM-CM gratings compete, binocular rivalry dynamics only follow the Levelt’s first and second laws (Fig. 3I–L). LM stimuli (i.e. gratings constructed from luminance-modulated dynamic noise) do follow the predictions of Levelt’s four laws.

When CM gratings compete with orthogonally orientated LM gratings, a mild, but not significant increase of flip rate was observed (Fig. 3H) with increases in stimulus visibility from 3.5 to 7x detection threshold. One could argue that if CM’s visibility was further increased, this could result in a significantly increased flip rate, in agreement with Levelt’s fourth law. The predominance of perception of CM over LM stimuli, and the observation that superimposition tends to increase with increasing visibility, however, would argue against this notion. It is noteworthy that the flip rate tends to increase when 3.5x and 7x LM stimuli rival (Fig. 3K), which is not in line with the third law. A condition in which 3.5x versus 43x LM gratings was tested and found to follow Levelt’s third law. A summary of results about whether or not Levelt’s laws hold for LM-LM, CM-CM and LM vs. CM rivalling gratings is provided in Table 1.

**Table 1:** Comparison of whether Levelt’s four laws hold for various stimulus types.

*Levelt’s laws*	L vs. L (see Brascamp *et al*. 2015)	LM vs. LM (Results of this paper)	CM vs. CM (Results of this paper)	CM vs. LM (Results of this paper)
***First***	✓	✓	✓	X
***Second***	✓	✓	✓	X
***Third***	✓	✓	X	X
***Fourth***	✓	✓	X	X

The check marks indicate that the laws hold for the specific conditions whereas the crosses indicate that the law does not hold. L indicates noiseless luminance modulated stimuli.

**Figure 5:**
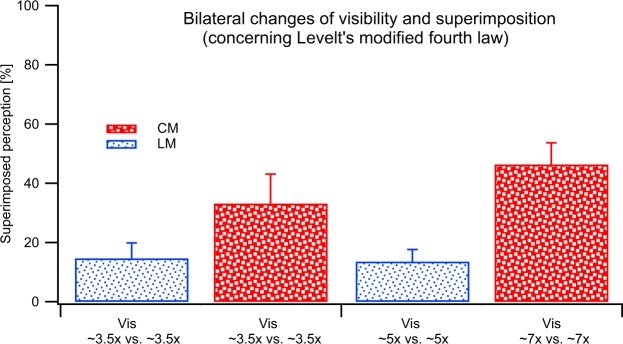
Proportions of superimposition for the CM vs CM (red, big dotted bars) and LM vs LM (blue small dotted bars) stimulus conditions. The results were averaged across trials and participants. The error bars represent +1SEM.

Surprisingly, CM stimuli tend to generate greater proportions of superimposition when the visibility levels of both eyes equally increase (see Fig. 5). There is a significant interaction between stimulus type (LM-LM versus CM-CM) and visibility level [*p* < 0.001]. This result implies that an increase of bilateral visibility level or ‘stimulus strength’ for CM stimuli increases binocular combination rather than exclusive visibility and binocular rivalry alternation, which is the opposite of findings that were generated by luminance defined stimuli^37^.

## Conclusions

The current study shows that highly visible LM gratings do not predominate perception over less visible orthogonally orientated CM gratings under binocular rivalry conditions. When CM-CM gratings compete with each other, only the first two laws are correct in predicting binocular rivalry characteristics. Also, Levelt’s four laws, all of which hold for L-only^2^ and LM-LM rivalling gratings, do not hold when LM and CM gratings compete. We discussed three potential explanations for the current results, namely visual saliency, asymmetric feedback, or a combination of both.
